# Unveiling new features of the human pathogen *Cryptococcus neoformans* through the reconstruction and exploitation of a dedicated genome-scale metabolic model

**DOI:** 10.1016/j.csbj.2025.05.034

**Published:** 2025-05-23

**Authors:** Romeu Viana, Diogo Couceiro, William Newton, Luís Coutinho, Oscar Dias, Carolina Coelho, Miguel Cacho Teixeira

**Affiliations:** a Department of Bioengineering, Instituto Superior Técnico, University of Lisbon, Lisboa 1049-001, Portugal; b iBB - Institute for Bioengineering and Biosciences, Associate Laboratory Institute for Health and Bioeconomy - i4HB, Lisboa 1049-001, Portugal; c INESC-ID, R. Alves Redol, 9, Lisbon 1000-029, Portugal; d MRC Centre for Medical Mycology at University of Exeter, University of Exeter, Exeter, United Kingdom; e CEB - Centre of Biological Engineering, Universidade do Minho, Braga 4710-057, Portugal

**Keywords:** *C. neoformans*, Global stoichiometric model, Drug targets, Metabolic features, Neurotropism

## Abstract

*Cryptococcus neoformans* is notorious for causing severe pulmonary and central nervous system infections, particularly in immunocompromised patients. High mortality rates, associated with its tropism and adaptation to the brain microenvironment and its drug resistance profile, make this pathogen a public health threat and a World Health Organization (WHO) priority. This study presents the first reconstructed genome-scale metabolic model (GSMM), iRV890, for *C. neoformans var. grubii*, which comprises 890 genes, 2598 reactions, and 2047 metabolites across four compartments. The GSMM iRV890 model was reconstructed using the open-source software tool merlin 4.0.2, is openly available in the well-established systems biology markup language (SBML) format and underwent validation using experimental data for specific growth and glucose consumption rates, and 222 nitrogen and carbon assimilation sources, with a 85 % prediction rate. Based on the comparison with GSMMs available for other pathogenic yeasts, unique metabolic features were predicted for *C. neoformans*, including key pathways shaping dynamics between *C. neoformans* and human host, as well as its underlying adaptions to the brain environment. Finally, the 96 predicted essential genes from the validated model are investigated as potential novel antifungal drug targets—including Erg4, Chs1, Fol1, and Fas1—which represent promising candidates for targeted drug development due to their absence in human cells.

## Graphical Abstract

**Figure:**
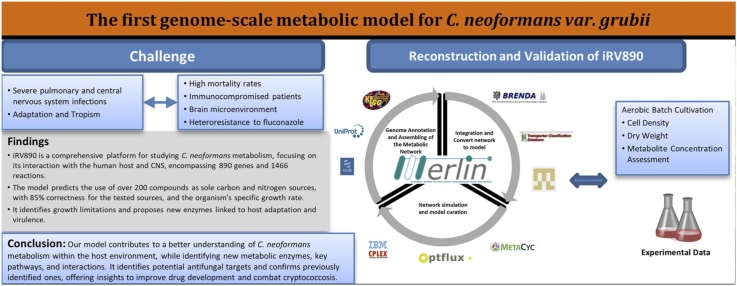

## 1 Introduction

Cryptococcal meningitis is a disease caused by a few pathogenic basidiomycetous yeast species, namely *Cryptococcus neoformans* (*C. neoformans*) and *Cryptococcus gatii*. Three *Cryptococcus* species/variants cause cryptococcosis: *C. neoformans var. grubii* (serotype A), responsible for 95 % of Cryptococcus infections worldwide [1]; *C. neoformans var. neoformans* (serotype D) and *Cryptococcus gattii* (serotypes B and C) geographically restricted to tropical and/or subtropical regions [2].

These species are notorious for inducing severe pulmonary and central nervous system infections [3]. While these pathogens are harmless in healthy individuals, they pose a serious threat to immunocompromised patients, especially those with acquired immunodeficiency syndrome (AIDS) or those undergoing immunosuppressive therapies, causing severe meningoencephalitis and other serious neurological complications [4], [5], [6]. The latest systematic review, using data from more than 120 countries, estimates that cryptococcal meningitis affects 190000 people worldwide annually while being associated with a mortality rate of 76 % [7]. Cryptococcal infections are commonly treated with combination therapy, usually flucytosine with amphotericin B in the first induction stage, followed by consolidation and long-term maintenance with high-dose fluconazole [8]. Anti-cryptococcal monotherapy is regarded as non-optimal, as it carries the risk of drug resistance [9]. Yet, for the most part, due to limited drug access, fluconazole monotherapy is still used. An increase in fluconazole resistance among *C. neoformans* isolates was observed in past decades [10], [11]. Fluconazole resistance is particularly notorious in isolates from relapse disease [12]. Despite verified in vitro susceptibility, echinocandins are not used clinically to treat cryptococcosis due to intrinsic resistance in vivo [13], attributed to their inability to penetrate the blood-brain barrier. Another possible contributor to echinocandin resistance in *Cryptococcus* species is fungal cell wall melanization through the action of a fungal laccase, which uses the L-DOPA and dopamine found in the human brain as precursors [14]. Melanin is an important virulence factor in *C. neoformans* since it can neutralize oxidative stress radicals [15] and toxic compounds, including some antifungal drugs, such as caspofungin and amphotericin B [16], [17].

*Cryptococcus neoformans* is widely spread in the environment, with worldwide distribution, in bird guano, soils and trees. Fungal particles are then inhaled by humans and other mammals [2]. This pathogen is known for its high resistance to harsh environments in nature and in mammalian hosts [18], and after inhalation into the host lungs, *Cryptococcus* can stay in a dormant latent granulomatous form for a long time [3]. However, tropism for the central nervous system is not yet fully understood [2], [3]. Despite being a public health threat and a WHO priority pathogen [19], *C. neoformans* still has many aspects of its peculiar metabolism associated with the central nervous system and interactions with the host that remain poorly understood [20].

Genome-scale metabolic models (GSMMs) are computational frameworks that reconstruct the full metabolic network of an organism based on its annotated genome, biochemical data, and physiological information [21]. These models consist of reactions, metabolites, and genes and are commonly represented in stoichiometric matrices that facilitate the simulation of metabolic fluxes through constraint-based modelling techniques such as Flux Balance Analysis (FBA). GSMMs provide a system-level view of metabolism, allowing researchers to predict cellular behaviour under various environmental and genetic conditions, explore metabolic capabilities, and design metabolic engineering strategies [22].

The development of GSMMs began in the early 2000s with the reconstruction of the first comprehensive model for *Haemophilus influenzae* [23], followed by other organisms including bacteria and yeast. Historically, GSMMs have been primarily used in the metabolic engineering of microbial cell factories, owing to their ability to simulate global metabolic behaviour and guide the optimization of value-added compound production [24]. Since then, advances in genomics, bioinformatics, and computational tools have led to a rapid expansion in the number and accuracy of GSMMs across diverse organisms, including pathogenic fungi, and have enabled systems-level insights into host-pathogen interactions, drug target identification, and metabolic adaptations associated with survival and virulence [25], [26], [27], [28], [29], [30].

This work presents iRV890, the first reconstructed GSMM for the human pathogen *C. neoformans var. grubii*, a frequent variant of these pathogenic species, is presented. The model is provided in the widely used SBML format to facilitate other researchers' usage. Model validation included comparison with experimental data for nitrogen and carbon assimilation from phenotypic arrays covering 222 sources [31]. Specific growth and glucose consumption rates were experimentally quantitatively determined to validate the model's predictive power. A set of essential genes derived from the validated model is predicted and discussed regarding their potential as novel antifungal drug targets.

Additionally, a comparison with GSMMs from other pathogenic yeast species and *S. cerevisiae* was performed to evaluate gene essentiality predictions and identify unique metabolic features of *C. neoformans*. Based on our findings, there is also discussion about some peculiar characteristics and pathways of this fungus relevant to its pathogenicity. The iRV890 model provides a promising platform for global elucidation of the metabolic features of *C. neoformans var. grubii*, with an expected impact in guiding the identification of new drug targets and understanding the complex metabolism of this pathogen in the context of the human brain.

## 2 Materials and methods

### 2.1 Model development

The genome-scale metabolic model of *C. neoformans var. grubii H99*, designated as iRV890, was reconstructed using *merlin* 4.0.5 [32] following the methodology described elsewhere [33] and further detailed in the 2.2, 2.3, 2.4, 2.5, 2.6. OptFlux 3.0 [34] was then used for curation and the subsequent validation stages. The IBM CPLEX 12.10 solver executed all computational analyses. The model is provided in SBML format in Supplementary Data 2 and can be exploited, for example, using the merlin platform, which offers a range of tools for simulation and gene essentiality analysis. A step-by-step tutorial detailing how to download merlin, import the model into merlin, and utilize the aforementioned tools is available at: https://merlin-sysbio.org/documentation/.

### 2.2 Genome annotation and assembling of the metabolic network

The genome sequence of the *C. neoformans var. grubii* and the Taxonomy ID 235443 were retrieved from the NCBI Assembly database, with the accession number ASM1180120v1 [35] and from the NCBI Taxonomy database [36], respectively. The genome-wide functional annotation was based on the taxonomy and frequency of similar sequences through remote DIAMOND alignment [37] and similarity searches using the UniProtKB/Swiss-Prot database. Draft network assembly relied on protein-reaction associations available in the Kyoto Encyclopedia of Genes and Genomes (KEGG) database [38], with all reactions categorized as spontaneous or non-enzymatic also incorporated in the initial draft model. Hit selection was performed as described elsewhere [33], and phylogenetic proximity was implemented based on a phylogenetic tree from the literature [32]. This process was automated via the “Automatic workflow” *merlin* tool and then integrated into the draft model [32].

### 2.3 Reversibility, directionality and balancing

Reaction reversibility and stoichiometry curation involved a multi-step process combining automated and manual efforts. Initially, *merlin* assisted in correcting the direction and reversibility of reactions, utilizing references from remote databases like eQuilibrator [39] to predict reaction directionality as described by Dias *et al.* [33]. To ensure that all reactions in the network are balanced, the authors performed extensive manual curation, exploiting databases such as MetaCyc [40], Brenda [41], UniProt [42], FungiDB [43], RHEA [44], KEGG [38] and existing literature, and with the correct directionality. Supplementary Data 1 includes all manually edited reactions.

### 2.4 Compartmentalization and transport reactions

This model includes four compartments: extracellular, cytoplasm, mitochondrion, and peroxisome and one intercompartment, the cytoplasmic membrane. The compartments for each enzyme were predicted using the DeepLoc - 2.0 [45] and directly imported to *merlin*. The transport reactions were automatically generated by TranSyT [46], a tool integrated in *merlin*, based on the public database TCDB [47]. Additional transport reactions across internal and external membranes for common metabolites, such as H_2_O, CO_2_, and NH_3_, often carried out without a transporter, were added to the model with no gene association.

### 2.5 Biomass equation

The biomass formation, depicted through an equation including proteins, DNA, RNA, lipids, carbohydrates, and cofactors, details the composition information for each macromolecule sourced from literature or experimental data. All calculations were performed as described in previous methodology [48] and are detailed in Supplementary Data 1. ATP requirements for biomass production and growth-associated maintenance (GAM) were added to the biomass equation with a value of 25.65 mmol ATP/gDCW, based on the ATP requirements for the biosynthesis of cell polymers as reported in [49], and ATP requirements for non-growth-associated maintenance (NGAM) was inserted in the model by an equation with specific fixed flux boundaries inferred from *Candida tropicalis* [49]. The theoretical phosphorus-to-oxygen ratio used in the *S. cerevisiae* iMM904 metabolic model was applied to our model, adding three generic reactions contributing to this ratio:

**Reaction R00081:**

(1) 1.0 Oxygen_mito_ + 4.0 Ferrocytochrome c_mito_ + 6.0 H^+^_mito_ ↔ 2.0 H2O_mito_ + 4.0 Ferricytochrome c_mito_ + 6.0 H^+^_cyto_

**Reaction R_Ubiquinol_Cytochrome_Reductase:**

(2) 1.0 Ubiquinol_mito_ + 2.0 Ferricytochrome c_mito_ + 1.5 H^+^_mito_ ↔ 1.0 Ubiquinone_mito_ + 2.0 Ferrocytochrome c_mito_ + 1.5 H^+^_cyto_

**Reaction T_ATP_Synthase:**

(3) 1.0 Orthophosphate_mito_ + 1.0 ADP_mito_ + 3.0 H^+^_cyto_ ↔ 1.0 ATP_mito_ + 1.0 H2O_mito_ + 3.0 H^+^_mito_

**The final balance reaction:**

(4) 3.0 Orthophosphate_mito_ + 1.0 Oxygen_mito_ + 3.0 ADP_mito_ + 2.0 Ubiquinool_mito_ ↔ 3.0 ATP_mito_ + 5.0 H_2_O_mito_ + 2.0 Ubiquinone_mito_

### 2.6 Network simulation and model curation

Extensive manual curation was needed to correct gaps in some pathways during the model reconstruction process due to incorrect reversibility, incomplete reactions, annotation errors, and blocked metabolites. Each case was meticulously inspected and studied, and reactions were edited, manually added to, or removed from the model based on evidence from the literature or deposited on databases such as KEGG pathways, MetaCyc, and FungiDB. The detailed list of all alterations is provided in Supplementary Data 1.

This process used *merlin*'s “Find blocked reactions” feature to support and accelerate the reconstruction. Additionally, *BioISO*, a tool based on the Constraints-Based Reconstruction and Analysis (COBRA) and Flux Balance Analysis (FBA) [50] frameworks, which is also integrated within *merlin*, identified potential network errors and further accelerated the gap-filling process.

### 2.7 Model validation

#### 2.7.1 Strains and growth media

*Cryptococcus neoformans var. grubii* H99E strain, from the laboratory of Jennifer Lodge, was obtained from the Fungal Genomic Stock Center and routinely maintained in Yeast extract–Peptone–Dextrose (YPD), containing 20 g/L glucose (Merck, Darmstadt, Germany), 20 g/L peptone (Merck, Darmstadt, Germany), and 10 g/L yeast extract (Merck, Darmstadt, Germany). The parental KN99 and derived KN99_ΔCNAG_02553 were obtained from the deletion library created by the Madhani laboratory [51] through the Fungal Genetics Stock Center and grown on YNB medium, containing 1.7 g/L Yeast Nitrogen Base, without amino acids (Difco BD, England, United Kingdom) and 20 g/L inositol, used as carbon source. Synthetic minimal media (SMM), 20 g/L glucose (Merck, Darmstadt, Germany), 2.7 g/L ammonium sulphate (Merck, Darmstadt, Germany), 0.05 g/L magnesium sulphate (Riddle-de-Haen), 2 g/L potassium dihydrogen phosphate (Panreac, Barcelona, Spain), 0.5 g/L calcium chloride (Merck, Darmstadt, Germany), and 100 µg/L biotin (Sigma), was used for batch cultivation experiments used to validate model predictions.

#### 2.7.2 Aerobic batch cultivation

*Cryptococcus neoformans var. grubii* was batch cultivated in SMM or YNB medium. Exponential phase inocula were prepared with an Optical Density (OD) of 0.3 at 600 nm (Hitachi u2001), and cells were transferred to Erlenmeyer flasks containing 250 mL of fresh medium and cultivated at 30 ºC with orbital agitation (250 rpm) for the duration of the experiment.

#### 2.7.3 Cell density, dry weight, and metabolite concentration assessment

Throughout cell cultivation in SMM, 4 mL samples were collected every two hours for subsequent quantification of biomass and extracellular metabolites. Cell density was monitored by measuring OD600nm. For dry weight determination, culture samples were centrifuged at 13,000 rpm for 3 minutes, and the resulting pellets were freeze-dried for 72 hours at −80 ºC before being weighed. Extracellular metabolites, including glucose, ethanol, glycerol, and acetic acid, were identified and quantified by High-performance liquid chromatography (HPLC) on an Aminex HPX-87H Ion Exchange chromatography column, eluted with 0.0005 M H_2_SO_4_ at a flow rate of 0.6 mL/min at room temperature. Samples were analyzed in triplicate, and concentrations were determined using appropriate calibration curves. According to the manufacturer (Bio-Rad), the Aminex HPX-87H ion exchange column is optimized for analysing carbohydrates in solution with carboxylic acids, volatile fatty acids, short-chain fatty acids, alcohols, ketones, and neutral metabolites, making it ideally-suited for fermentation monitoring, biological fluid analysis, and simultaneous profiling of monosaccharides and organic acids. During the exponential growth phase, the specific growth rate, glucose consumption rate, and production rates of ethanol, glycerol, and acetic acid were calculated as described elsewhere [52].

#### 2.7.4 Network simulation and analysis

All the phenotypic simulations were performed with Flux Balance Analysis (FBA) in OptFlux 3.0 using the IBM CPLEX solver, including gene and reaction essentiality, growth assessment, metabolite production and consumption, and carbon and nitrogen source utilization. For gene and reaction essentiality, *in silico* growth was simulated in environmental conditions mimicking RPMI medium and a biomass flux lower than 5 % of the wild-type strain was considered the threshold for essentiality after the respective gene/reaction knockout. Gene and reaction knockout was simulated by restraining its corresponding flux bounds to zero.

## 3 Results and discussion

### 3.1 Model characteristics

The *C. neoformans var. grubii* genome-scale metabolic model reconstructed herein and denominated iRV890, which comprises 890 genes associated with 2598 reactions, of which 683 correspond to transport reactions, and 2047 metabolites across four compartments (extracellular, cytoplasm, mitochondria, and peroxisome). The model is provided in SBML format in Supplementary Data 2. Among the 2598 reactions, 1747 are cytoplasmic, 351 mitochondrial, 60 peroxisomal, and 440 are drains (exchange constraints used to simulate the import of media components or the leakage or export of extracellular metabolites).

During the manual curation process, a total of 639 reactions/genes required alterations, including 80 that were mass balanced, 518 that were corrected for reversibility, directionality, or added or removed from the model, and 41 whose annotation was corrected, as detailed in Supplementary Data 1.

The Biomass equation (Table 1) encompasses the cell's major components and their respective and relative contributions, including DNA, RNA, lipids, carbohydrates, and cofactors. The equation's composition in carbohydrates [53] and lipids [54], [55], [56] was inferred from literature data for *C. neoformans*. The composition of proteins, DNA and RNA was determined by the e-BiomassX tool, where the whole genome sequence was used to estimate the amount of each deoxyribonucleotide as described in [57]. Total RNA in the cell was estimated using mRNA, rRNA, and tRNA as described in [24], [57].

**Table 1:** Biomass composition used in the model iRV890. The complete individual validated contributions of each metabolite are shown in Supplementary Data 1, alongside the literature references used to substantiate the metabolite composition in *C. neoformans*. When *C. neoformans* data was unavailable, data from other yeast species was used as a proxy.

**Metabolite**	**g/gDCW**		**Metabolite**	**g/gDCW**
				
**Lipids**			**Proteins**	
Lanosterol	0.000122		L-Valine	0.019058
Zymosterol	0.000254		L-Tyrosine	0.020501
Squalene	0.000209		L-Tryptophan	0.006392
Ergosterol	0.000724		L-Threonine	0.022013
Phosphatidylserine	0.005024		L-Serine	0.027696
Phosphatidylinositol	0.004638		L-Proline	0.015390
Phosphatidylcholine	0.031241		L-Phenylalanine	0.022928
Phosphatidylethanolamine	0.017714		L-Methionine	0.008274
Cardiolipin	0.002254		L-Lysine	0.033668
Phosphatidic acid	0.000644		L-Leucine	0.036895
Phosphatidylglycerol	0.000644		L-Isoleucine	0.028492
Tetradecanoic acid	0.000020		L-Histidine	0.010159
Hexadecanoic acid	0.000097		L-Glutamate	0.029371
Octadecanoic acid	0.000038		L-Cysteine	0.003902
Dodecanoic acid	0.000021		L-Aspartate	0.023883
Decanoic acid	0.000011		L-Asparagine	0.027060
Octanoic acid	0.000038		L-Arginine	0.020979
Octadecanoic acid	0.000038		L-Alanine	0.012706
(9*Z*)-Octadecenoic acid	0.000093		Glycine	0.010258
(9Z,12Z)-Octadecadienoic acid	0.000116		L-Glutamine	0.020550
(9Z,12Z,15Z)-Octadecatrienoic acid	0.000002			
Triacylglycerol	0.032969		**Soluble Pool**	
Sterol esters	0.001127		Pyridoxine 5′-phosphate	0.000833
			FAD	0.000833
**Carbohydrates**			Thiamine(1 +) diphosphate	0.000833
Chitin	0.005645		NAD	0.000833
Mannan	0.033956		Glutathione	0.000833
β (1,3)-Glucan	0.360399		Riboflavin	0.000833
			Eumelanin	0.000833
**Ribonucleotides**			Ubiquinone−6	0.000833
UTP	0.006713		NADP	0.000833
GTP	0.006806		COA	0.000833
CTP	0.005381		FMN	0.000833
ATP	0.007101		5-Methyltetrahydrofolate	0.000833
				
**Deoxyribonucleotides**				
dTTP	0.016718			
dGTP	0.017029			
dCTP	0.015059			
dATP	0.017193			

The translated genome sequence was used to calculate the amino acid composition using the percentage of each codon usage, as described in [57]. Essential metabolites were included in the biomass composition to qualitatively account for the essentiality of their synthesis pathways [58], [59]. The growth and non-growth ATP requirements were adopted from *S. cerevisiae* [60].

### 3.2 Model validation

#### 3.2.1 Carbon and nitrogen source utilization

*In silico* simulations were conducted using 222 compounds as the exclusive carbon or nitrogen sources under conditions mimicking those of the minimal medium reported in [31]. The in *silico* growth was compared to publicly available phenotypic microarray (Biolog platform) data for *C. neoformans var. grubii* performed in [31]. Base-level validation of the GSMM in minimal medium was deemed sufficient to guarantee connectivity, as it forced biomass production from the most fundamental metabolic precursors. In these conditions, validation relies on the model’s ability to reproduce experimentally observed behaviours, such as glucose consumption and ethanol production, which were included in the model and have topologically direct relationships within the network. These quantifiable, primary metabolites ensure that the core structure and function of the model behave as expected, thereby validating the model without the need to include additional complexity beyond central metabolism. A total of 155 sole carbon sources and 67 sole nitrogen sources were evaluated. For the analyses, stationary phase yeast conditions data was used after calculating the difference from the respective negative control group without any carbon or nitrogen sources. The iRV890 model correctly predicted growth in 85 % (133/155) of the carbon sources tested and 85 % (57/67) of the nitrogen sources (Supplementary Table 1).

In some cases of failed predictions, such as L-ornithine, glycerol (carbon source), amino acids or D-Glucosamine (nitrogen source), genetic information and the model include all the necessary steps to predict their assimilation as sole carbon/nitrogen sources, but no growth was experimentally observed. In such cases, the failed prediction may be related to non-metabolic factors not considered in model simulations or due to inaccuracies regarding the annotation of transporters, which is still a big challenge in the current model development process [61]. In other cases, however, the prediction model failed because specific enzymes are not yet characterized for *C. neoformans* despite growth in experimental conditions. Although the underlying genes and proteins are still unidentified, the comparison between the model predictions and experimental evidence suggests that the following enzyme activities are likely to be present in *C. neoformans*: 1.2.1.3 (aldehyde dehydrogenase), 1.1.1.21 (aldose reductase), 3.1.1.65 (L-rhamnono-1,4-lactonase), 1.1.1.56 (ribitol 2-dehydrogenase), 5.1.3.30 (D-psicose 3-epimerase), 2.7.1.55 (allose kinase), 4.1.2.10 ((R)-mandelonitrile lyase), 5.3.1.3 (D-arabinose isomerase), 3.2.1.86 (6-phospho-beta-glucosidase), 4.1.2.4 (deoxyribose-phosphate aldolase), 3.2.1.86 (6-phospho-beta-glucosidase) and 1.1.1.16 (galactitol 2-dehydrogenase). Identifying and characterizing these predicted functions and their associated genes will enhance our understanding of specific carbon and nitrogen assimilation pathways for *C. neoformans*, potentially uncovering novel virulence mechanisms linked to host adaptation. Therefore, further investigation into these enzymes represents a valuable direction for future research. Altogether, the model achieved 85 % predictability, which is a high value, especially considering that the extensive list of carbon and nitrogen sources tested includes many that are not commonly used in traditional metabolic and phenotypic experiments and thus lack biochemical characterization.

#### 3.2.2 Growth parameters in batch culture

To quantitatively validate the model, the specific growth rate, glucose consumption rate, and metabolite production rates were experimentally determined and compared with in silico predicted values. For a glucose consumption rate of 1.72 mmol.gDCW^−1^.h^−1^, a specific growth rate of 0. 188 h^−1^ was experimentally determined, leading to no detectable ethanol, glycerol, or acetate production. For model validation purposes, the system behaviour was simulated *in silico* using SMM medium and a fixed glucose uptake flux of 1.72 mmol.g^−1^ dry weight.h^−1^, matching the experimentally determined value. Other nutrient fluxes were left unconstrained, as the system was glucose-limited under these conditions. The simulation predicted a specific growth rate of 0.128 h^−1^, which differs only in 0.06 h^−1^ from the experimentally determined value (Table 2). In these conditions, the model did not predict the formation of glycerol, acetic acid, or ethanol as by-products, which is consistent with the experimental data. Moreover, the model is accurate at predicting no growth of *C. neoformans* under anaerobic conditions, an expected observation since this pathogen is an obligate aerobic fungus [62].

**Table 2:** Growth parameter values predicted by the iRV890 model and comparison with those determined experimentally.

	Specific growth rate (h^−1^)	q (mmol g^−1^ dry weight h^−1^)			
		Glucose	Ethanol	Glycerol	Acetic acid
*In silico*	0.128	1.72	0	0	0
*In vitro*	0.188 ± 0.025	1.72	0	0	0

### 3.3 *C. neoformans* unique metabolic features

**Fig. 1:**
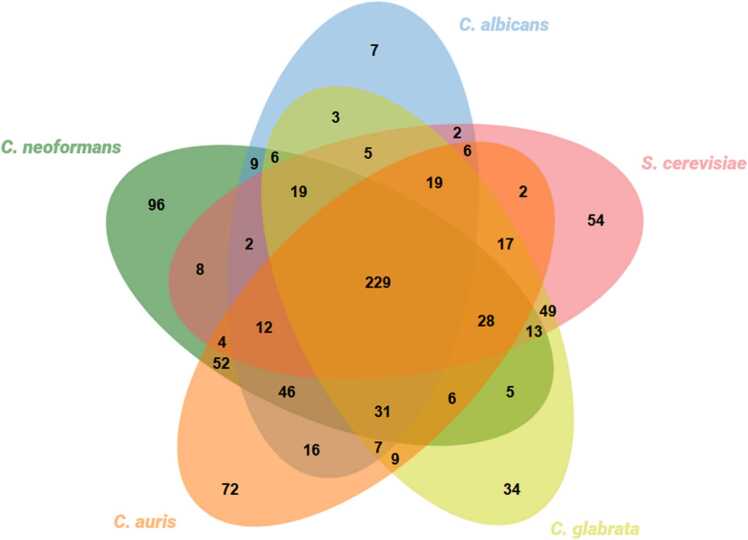
Multi-species comparison in terms of proteins with an associated EC number present in the *C. neoformans* iRV890, *C. albicans* iRV781, *C. auris* iRV973, *S. cerevisiae* iIN800 and *C. glabrata* iNX804 GSMMs. The multiple intersection was performed using jvenn [65].

The *C. neoformans* GSMM was compared with previously built models for *Candida glabrata* [58], *Candida albicans* [30], *Candida auris* [63] and *S. cerevisiae* [64] by others and us to uncover unique metabolic features of this pathogen. A comparison across the existing models was carried out based on shared EC numbers. After intersecting the EC numbers in each of the five models, 40 % (229/566) of the EC numbers were the same among all the tested yeasts (Fig. 1). Additionally, the remaining 17 % (96/556) were exclusive to the *C. neoformans* model and may represent unique metabolic features of this species relative to the remaining. We confirmed that none of these 96 EC numbers were associated with outdated, incomplete or incorrect reaction associations. However, a small subset of these 96 EC numbers may be present in other species included in the comparison but not accounted for in their respective GSMMs during reconstruction.

The metabolic features or pathways relevant in the context of fungal infection in the host brain were searched manually from the list of 96 unique EC numbers found for *C. neoformans* (Supplementary Data 1) and compared to extant knowledge of these pathways being defence mechanisms, or enabling host adaptation, through degradation or biosynthesis of specific metabolites. A few of these unique EC numbers with a higher potential of impacting *C. neoformans* pathogenesis are discussed below:

L-arabinitol 4-dehydrogenase and D-arabinitol dehydrogenase (1.1.1.12 and 1.1.1.287, respectively) are two enzymes that are required for L-arabinitol assimilation as a carbon source, which is a particular metabolic feature of *C. neoformans* when compared to other yeast species (Supplementary Table 1). Indeed, neither *Candida* species [30], [66] nor *S. cerevisiae* [67] can assimilate L-arabitol unless genetically engineered [68]. Interestingly, environment isolates containing SNPs in the PTP1 gene, encoding a *C. neoformans* arabitol transporter, were associated with increased patient survival, while a virulence defect was observed in BALB/c mice due to PTP1 gene deletion [69]. PTP1 expression was also highly induced in macrophage and amoeba infection [70]. Since arabitol is present in the cerebrospinal fluid [71], this pathway might feed from polyols in CNS and contribute to explaining the brain tropism of *C. neoformans*, compared to other fungal species.

**Fig. 2:**
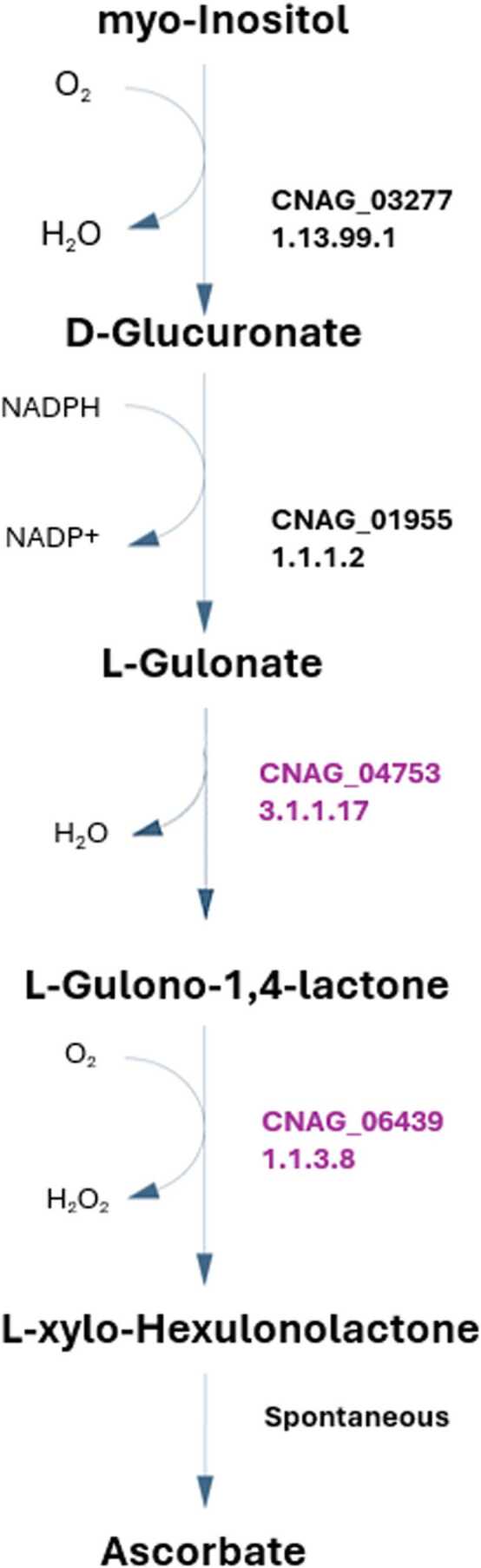
*C. neoformans* pathway for ascorbate biosynthesis, with the respective *C. neoformans var. grubii* EC numbers in the iRV890 model. The 1.1.3.8 and 3.1.1.17 enzymes, unique to *C. neoformans* among other pathogenic yeasts, are highlighted in purple.

L-gulonolactone oxidase and gluconolactonase (1.1.3.8 and 3.1.1.17, respectively) are two enzymes that participate in ascorbate metabolism, allowing the utilization of Inositol and D-glucuronate as the source for L-ascorbate biosynthesis (Fig. 2). Interestingly, two independent studies reported that ascorbate, an antioxidant, lowers the susceptibility towards fluconazole in *C. neoformans* [72], [73]. However, this effect seems unrelated to its antioxidant role but with ascorbate-induced up-regulation of Upc2, a transcriptional regulator of genes involved in ergosterol biosynthesis, as shown in *C. albicans* [74]. The ability of *C. neoformans* to synthesize ascorbate from inositol is particularly noteworthy, given the abundance of inositol in the human brain [20] and the widespread use of fluconazole in treating infections. Further, ascorbate possibly contributes to resistance to ROS. Having a mechanism to produce a compound that mitigates the toxicity of fluconazole and ROS could contribute to a significant adaptive advantage for this species.

**Fig. 3:**
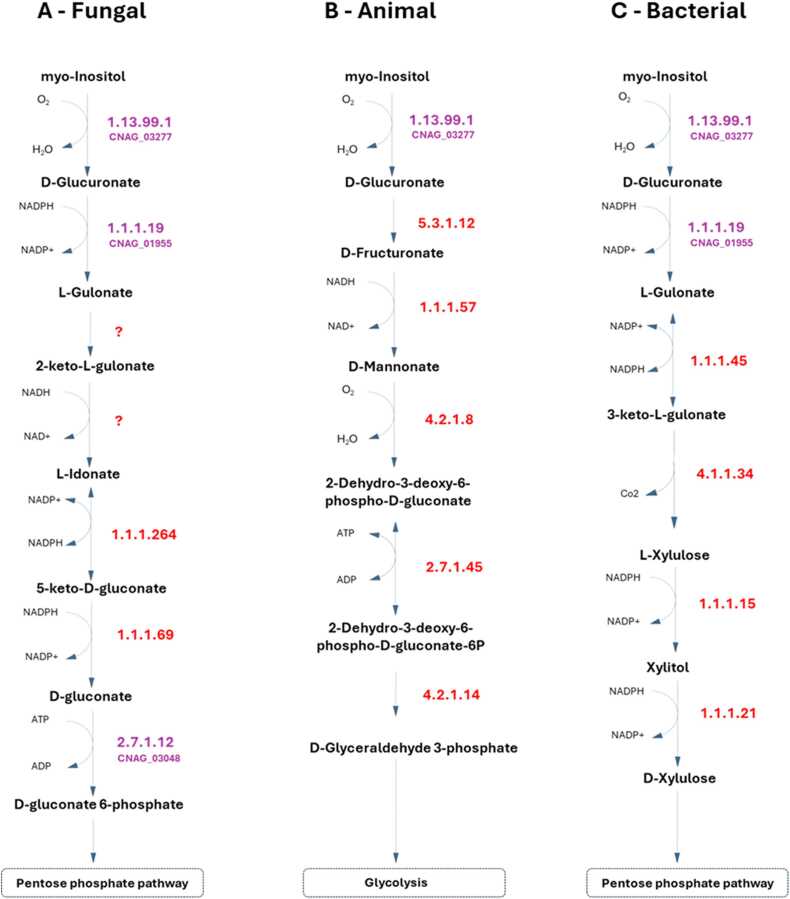
Metabolic pathways for inositol assimilation as carbon source, A – based on the proposed fungal inositol assimilation pathway reported in Kuivanen et al# 2016 [75], B – based on the animal inositol assimilation pathway, and C – based on the bacterial inositol assimilation pathway. The respective *C. neoformans var. grubii* genes in iRV890 are highlighted in purple, the currently unknown genes are in red, and the proposed reactions with an unknown EC number are represented as a question mark in red.

Furthermore, the 1.1.3.8 and 3.1.1.17 enzymes are also significant for inositol assimilation as a carbon source through variation of the previous pathway. This pathway was suggested recently as an alternative pathway in fungi for inositol assimilation, and since inositol is highly abundant in the human brain, this may represent a crucial metabolic feature for *C. neoformans*. Two of the reactions reported were recreated to implement this pathway in the model and attributed with the names R2_Inositol_Pathway and R1_Inositol_Pathway, although the corresponding EC numbers and genes have not been identified in the annotated *C. neoformans* genome [75]. This pathway was exclusively recreated from literature, and while it lacks validation studies, two possible genes were hypothesized as probable candidates for encoding the 1.1.1.69 enzyme, CNAG_02553 and CNAG_00126, predicted by OrthoMCL [76]. Additional pathways for inositol assimilation are reported for animals (Fig. 3.B) and bacteria (Fig. 3.C); however, since *C. neoformans* lacks almost all the enzymes in those pathways, we considered that the newly reported one in fungi [75] was the most probable to occur in this pathogen. Taking advantage of the available ΔCNAG_02553 deletion strain, we tested whether a strain deleted for this putative enzyme could be grown in inositol as a single carbon source compared to the parental strain. However, regardless of the CNAG_02553 gene, *C. neoformans* can utilize inositol as the sole carbon source in SMM (YNB, supplemented with glucose or inositol; data not shown). Eventually, it would be necessary to knock out both CNAG_00126 and CNAG_02553 genes to obtain a strain unable to grow in media containing inositol as the sole carbon source. Further scrutiny is required to address this issue.

L-rhamnose 1-dehydrogenase (1.1.1.377) is required for L-rhamnose assimilation as the sole carbon source. Rhamnose is used by some pathogens, for example, *Pseudomonas aeruginosa*, to produce rhamnolipids. It constitutes a relevant virulence factor in those bacteria, with roles in biofilm formation, hydrophobic nutrient uptake, and host immunity evasion, characterized by increasing lung epithelial permeability [77], [78] and macrophage phagocytosis inhibition [79]. *Candida* species [30], [63], [66] and *S. cerevisiae* (unless engineered) [80] cannot assimilate L-rhamnose, and thus, assimilation of rhamnose is a particular metabolic feature of *C. neoformans* when compared to these yeast species (Supplementary Table 1).

Tetracycline 11a-monooxygenase (1.14.13.231) is an enzyme that allows the direct conversion of tetracycline into 11a-hydroxytetracycline, reported to confer resistance to all clinically relevant tetracyclines by efficient degradation of a broad range of tetracycline analogues. The hydroxylated product, 11a-hydroxytetracycline, is unstable and leads to intramolecular cyclization and non-enzymic breakdown to undefined products, completely neutralizing the tetracycline effects [81], [82]. Although tetracyclines are generally used as antibacterial antibiotics and have poor antifungal activity, the presence of this enzyme in *C. neoformans* should be considered when designing tetracyclines against fungi.

3-phytase (3.1.3.8) is an enzyme involved in inositol metabolism that may be involved in the production of phytic acid from inositol, a primary storage molecule of phosphorus and inositol in fungi (although not in the pathogenic *Candida* species), bacteria and plants [83]. Interestingly, this pathway has been shown to play a key role in *C. neoformans* virulence. Indeed, it was previously reported that deletion of the gene encoding the enzyme (EC number 2.7.1.158) immediately preceding 3-phytase leads to growth impairment and attenuated virulence in *C. neoformans*, associated with failed dissemination into the brain [84].

Hydroxyisourate hydrolase (3.5.2.17) is an enzyme essential for the assimilation of uric acid as the sole nitrogen source. Uric acid is a constitutive component of urine and bird guano. In bird guano, 70 % of the nitrogen is present in uric acid, with the rest consisting primarily of xanthine, urea, and creatinine [85]. Additionally, uric acid enhances the production of key cryptococcal virulence factors, including capsule and urease, an enzyme required for full fitness at mammalian pH and dissemination to the brain [86]. *C. neoformans* capsule is induced if uric acid is present [87].

L-tryptophan decarboxylase (4.1.1.105) catalyzes the conversion of L-tryptophan into tryptamine, which can then be converted into serotonin. It also shares structure with several aminergic neuromodulators. However, the reaction is bidirectional, and tryptamine can also be converted into L-tryptophan. While the role of this enzyme may be unclear in *C. neoformans*, it is potentially related to the brain environment, specifically in the utilization of serotonin as a nitrogen source, through its conversion to L-tryptophan.

DOPA decarboxylase, tyrosinase and laccase (4.1.1.28, 1.14.18.1, and 1.10.3.2, respectively) are particularly important in *C. neoformans*, as they are involved in the biosynthesis of melanin. Most fungi possess multiple melanin biosynthetic pathways, while *C. neoformans* exclusively synthesize melanin through the L-DOPA pathway [88]. Melanin can neutralize oxidative stress radicals as well as protect the pathogen against the host immune system, and antifungal drugs, such as caspofungin and amphotericin B. L-DOPA and Dopamine are present in the human brain and serve as precursors for dopamine biosynthesis in this pathogen. However, the reason for *C. neoformans* exclusively using this pathway is unclear compared to other human pathogenic fungi.

### 3.4 Drug target analysis based on gene essentiality prediction

Pathogen GSMMs are particularly useful for identifying potential new drug targets among predicted essential genes. For that purpose, the behaviour of the system simulation using RPMI medium, mimicking the environmental conditions of human serum, yielded a list of 157 enzymes and 101 genes predicted to be essential in *C. neoformans*. Among these targets, some have been previously identified as essential genes in other pathogenic yeasts (see Table 3), indicating potential drug targets common to all *Candida* species and *C. neoformans*. Notably, Erg11 and Fks1 are already targets of currently used antifungals, fluconazole and echinocandins, respectively. Additionally, Erg26, Erg27, Erg24, Erg4, Erg7, Erg12, and Erg13 have all been identified herein as potential drug targets within the ergosterol biosynthetic pathway. Erg4 is particularly interesting, as it lacks a human ortholog and may represent a superior candidate for designing compounds with enhanced selectivity and lower toxicity.

**Table 3:** Enzymes predicted to be essential in RPMI medium in 5 pathogenic fungal species, based on the screening of the genome-scale metabolic models of *C. neoformans* iRV890, *C. auris* IRV973, *C. parapsilosis* iDC1003, *C. albicans* iRV781, and *C. glabrata* iNX804. Grey rows highlight enzymes which are not encoded in the human genome. Data regarding the drug association was retrieved from the DrugBank database; only drugs with known pharmacological action against pathogens were selected**.**

***C. neoformans***	***C. albicans***		***C. glabrata***	***C. parapsilosis***	***C. auris***	***S. cerevisiae***	**Human**	**Pharmacological action**	**EC Number**	**Pathway/Target**
									
CNAG_04605	ERG26	CAGL0G00594g		CPAR2_302110	CJI97_000938	ERG26	NSDHL		1.1.1.170	Steroid
CNAG_00441	IMH3	CAGL0K10780g		CPAR2_104580	CJI97_000080	IMD4	IMPDH	-	1.1.1.205	Purine
CNAG_07437	ERG27	CAGL0M11506g		CPAR2_801560	CJI97_004310	ERG27	DHRS11	-	1.1.1.270	Steroid
CNAG_06534	HMG1	CAGL0L11506g		CPAR2_110330	CJI97_003299	HMG1	HMGCR	-	1.1.1.34	Terpenoid backbone
CNAG_00117	ERG24	CAGL0I02970g		CPAR2_405900	CJI97_003097	ERG24	TM7SF2	-	1.3.1.70	Steroid
CNAG_02830	ERG4	ERG4		ERG4	CJI97_002908	ERG4	-	-	1.3.1.71	Steroid
CNAG_04692	CDC21	CDC21		CPAR2_206550	CJI97_005101	TMP1	TYMS	-	2.1.1.45	Pyrimidine
CNAG_00700	ADE17	CAGL0A03366g		CPAR2_202250	CJI97_002511	ADE17	ATIC	-	2.1.2.3	Purine
CNAG_07373	URA2	CAGL0L05676g		CPAR2_203160	CJI97_002269	URA2	CAD	-	2.1.3.2	Pyrimidine
CNAG_06508	GSC1	FKS1		CPAR2_106400	FKS1	FKS1	-	Echinocandins	2.4.1.34	1,3-beta-glucan
CNAG_03196	URA5	URA5		CPAR2_802790	CJI97_002422	URA5	UMPS	-	2.4.2.10	Pyrimidine
CNAG_02853	ADE4	CAGL0M13717g		CPAR2_208260	CJI97_001833	ADE4	PPAT	-	2.4.2.14	Purine
CNAG_02084	BTS1	CAGL0H05269g		CPAR2_302840	CJI97_003197	BTS1	GGPS1	-	2.5.1.1	Terpenoid backbone
CNAG_07780	ERG20	ERG20		CPAR2_103950	CJI97_001757	ERG20	FDPS	-	2.5.1.10	Terpenoid backbone
CNAG_02787	C5_05130C	CAGL0F05555g		CPAR2_502760	CJI97_003836	CAB5	COASY	-	2.7.1.24	CoA
CNAG_02976	CR_03740C	CAGL0K11022g		CPAR2_202590	CJI97_005311	FMN1	RFK	-	2.7.1.26	Riboflavin
CNAG_02866	C6_02980C	CAGL0H01551g		CPAR2_602050	CJI97_004586	CAB1	PANK	-	2.7.1.33	CoA
CNAG_05935	URA6	CAGL0L09867g		CPAR2_105320	CJI97_000033	URA6	CMPK2	-	2.7.4.14	Pyrimidine
CNAG_06001	ERG8	ERG8		CPAR2_400710	CJI97_001215	ERG8	PMVK	-	2.7.4.2	Terpenoid backbone
CNAG_03335	C5_00260W	CAGL0D00550g		CPAR2_304260	CJI97_000019	PRS1	PRPS1	-	2.7.6.1	Purine
CNAG_05384	C4_05210W	CAGL0G03157g		CPAR2_500260	CJI97_005306	PIS1	CDIPT	-	2.7.8.11	Glycerophospholipid
CNAG_02795	ADE8	CAGL0F02761g		CPAR2_211620	CJI97_002826	ADE8	GART	-	2.1.2.2	Purine
CNAG_02609	COQ3	CAGL0I07601g		CPAR2_602300	CJI97_005452	COQ3	COQ3	-	2.1.1.114	Ubiquinone
CNAG_00138	COQ5	CAGL0J06710g		CPAR2_209250	CJI97_003704	COQ5	COQ5	-	2.1.1.201	Ubiquinone
CNAG_00040	ERG11	ERG11		ERG11	ERG11	ERG11	CYP51A1	Azoles	1.14.14.154	Steroid
CNAG_02844	PEL1	PGS1		CPAR2_805350	CJI97_000224	PEL1	PGS1	-	2.7.8.5	Glycerophospholipid
CNAG_02878	C6_01340C	CAGL0H04389g		CPAR2_602700	CJI97_005490	GEP4	PTPMT1	-	3.1.3.27	Glycerophospholipid
CNAG_00734	URA4	CAGL0J04598g		CPAR2_100500	CJI97_002941	URA4	CAD	-	3.5.2.3	Pyrimidine
CNAG_02294	ADE2	ADE2		CPAR2_805940	CJI97_004071	ADE2	PAICS	-	4.1.1.21	Purine
CNAG_04961	URA3	URA3		URA3	CJI97_003384	URA3	UMPS	-	4.1.1.23	Pyrimidine
CNAG_02786	FOL1	CAGL0J07920g		CPAR2_303390	CJI971_001274	FOL1	-	Sulfacetamide	4.1.2.25	Folate biosynthesis
CNAG_02786	FOL1	CAGL0J07920g		CPAR2_303390	CJI971_001274	FOL1	-	Sulfonamides	2.5.1.15	Folate biosynthesis
CNAG_05125	MVD	CAGL0C03630g		CPAR2_109530	CJI97_001340	MVD1	MVD	-	4.1.1.33	Terpenoid backbone
CNAG_00909	CAB3	CAGL0L05302g		CPAR2_800750	CJI97_003563	CAB3	PPCDC	-	4.1.1.36	CoA
CNAG_03270	ADE13	CAGL0B02794g		CPAR2_204960	CJI97_000801	ADE13	ADSL	-	4.3.2.2	Purine
CNAG_00265	IDI1	CAGL0J06952g		CPAR2_401630	CJI97_001183	IDI1	IDI1	-	5.3.3.2	Terpenoid backbone
CNAG_01129	ERG7	CAGL0J10824g		CPAR2_301800	CJI97_005090	ERG7	LSS	Oxiconazole	5.4.99.7	Steroid
CNAG_00143	ADE1	CAGL0I04444g		CPAR2_500190	CJI97_003065	ADE1	PAICS	-	6.3.2.6	Purine
CNAG_06314	ADE5,7	CAGL0H07887g		CPAR2_208400	CJI97_001704	ADE5,7	GART	-	6.3.3.1	Purine
CNAG_06314	ADE5,7	CAGL0H07887g		CPAR2_208400	CJI97_001704	ADE5,7	GART	-	6.3.4.13	Purine
CNAG_04192	ADE6	CAGL0K04499g		CPAR2_204070	CJI97_002160	ADE6	PFAS	-	6.3.5.3	Purine
CNAG_05759	ACC1	CAGL0L10780g		CPAR2_804060	CJI97_001038	ACC1	ACACA	-	6.4.1.2	Fatty acid
CNAG_02686	ERG12	CAGL0F03861g		CPAR2_803530	CJI97_005606	ERG12	MVK	-	2.7.1.36	Terpenoid backbone
CNAG_03311	ERG13	ERG13		CPAR2_701400	CJI97_004952	ERG13	HMGCS	-	2.3.3.10	Terpenoid backbone
CNAG_02099	FAS1	CAGL0D00528g		FAS1	CJI97_001309	FAS1	-	-	2.3.1.86	Fatty acid
CNAG_01877	GUA1	CAGL0F03927g		CPAR2_803560	CJI97_005609	GUA1	GMPS	-	6.3.5.2	Pyrimidine
CNAG_03099	CHS1	CAGL0I04818g		CPAR2_805640	CHS2	CHS2	-	-	2.4.1.16	Chitin
									

Similarly to *Candida*, which lacks a folate transporter [89] and relies on its *de novo* biosynthesis, *C. neoformans* seems to also lack a folate transporter, leading to the identification of Fol1, a multifunctional enzyme of the folic acid biosynthesis pathway, as a promising multi-yeast drug target. Furthermore, Fas1, a fatty acid synthase enzyme, and Chs1, a chitin synthase, also lack human orthologs and constitute promising alternative antifungal drug targets due to their relevant role in membrane and cell wall structure and integrity. Other noteworthy targets span various pathways, including purine metabolism, terpenoid backbone biosynthesis, pyrimidine metabolism, CoA biosynthesis, glycerophospholipid biosynthesis, and ubiquinone biosynthesis (Table 3). However, exploring these targets requires leveraging potential structural differences in the enzyme active site compared to their human counterparts. Since *C. neoformans* colonizes a different host environment and is phylogenetically distant from *Candida* spp., the evaluation extended to include potential new drug targets unique to this species and not shared by *Candida* spp. We identified only two such targets: the 1.14.18.1 tyrosinase, encoded by the gene CNAG_03009, and the 2.5.1.83 hexaprenyl diphosphate synthase, encoded by the gene CNAG_04375. While tyrosinase, responsible for melanin production, has a human ortholog (since humans also synthesize melanin via the L-DOPA), hexaprenyl diphosphate synthase (2.5.1.83) is fungal-specific and may represent an interesting target. This enzyme plays a crucial role in terpenoid backbone biosynthesis, serving as a key contributor to precursor synthesis for ubiquinone biosynthesis.

## 4 Conclusions

The construction and validation of iRV890, the first genome-scale metabolic model for *C. neoformans var. grubii*, is presented herein. iRV890 constitutes a robust platform for exploring and elucidating the metabolic features of this poorly understood pathogen, particularly concerning its interaction within the central nervous system and the human host. By encompassing 890 genes associated with 1466 reactions, this model offers a comprehensive view of the metabolic landscape of the pathogen. Through *in silico* simulations, we predicted the use of more than 200 compounds as sole carbon or nitrogen sources, and after comparison to experimental data from phenotypic microarrays, we gained valuable insights into the metabolic capabilities of *C. neoformans*. The model correctly predicts 85 % of the sole carbon and nitrogen sources tested. The model was able to approximately predict the specific growth rate of the organism and confirmed its inability to grow under anaerobic conditions or to accumulate glycerol, acetic acid, or ethanol as metabolic by-products during growth in SMM, with glucose as the carbon source. Additionally, we propose a list of yet unidentified enzymes expected to be present in *C. neoformans*, based on the carbon and nitrogen utilization and with the potential to represent new host adaptation or virulence mechanisms, including new clues on the pathway for inositol utilization in *C. neoformans*.

Our investigation into the unique metabolic features of *C. neoformans* has unveiled several pathways and enzymatic activities proposed to play pivotal roles in fungal infection within the host brain. Some enzymes constitute important virulence factors, such as tyrosinase and laccase, enzymes responsible for the production of melanin, which has an important role in host immune evasion [15], infection proliferation and drug resistance [16], [17]. Other enzymes are related to drug and stress resistance, such as tetracycline 11a-monooxygenase, L-gulonolactone oxidase and gluconolactonase. The remaining enzymes are directly related to alternative carbon/nitrogen source utilization and are important for environmental adaptation. For example, hydroxyisourate hydrolase is essential for uric acid assimilation as a nitrogen source, an important virulence factor mechanism, and 3-phytase is involved in inositol metabolism and storage, which is important for brain dissemination. Although beyond the scope of this study, it would also be interesting to explore the use of the reconstructed model in the prediction of secondary metabolites of relevance that may be produced in the brain environment, and validate these predictions through metabolomics analysis. It would also be important to evaluate the metabolic variability across C. neoformans clinical isolates, for example through genomic mining of biosynthetic gene clusters (BGCs), and compare it with virulence capacity.

In this work, we also propose several potential drug targets in *C. neoformans*. Notably, enzymes such as Erg4, Chs1, Fol1 and Fas1 present promising opportunities for targeted drug development due to their absence in human cells, offering opportunities for selective and low-toxicity compounds. The CNAG_03009 and CNAG_04375 genes, encoding a tyrosinase and a hexaprenyl diphosphate synthase, are presented as potential antifungal drug targets specific to *C. neoformans*. Further studies, in settings closely mimicking clinical conditions, are required to further validate the proposed targets. Additionally, they could provide key insights which may be leveraged to design and optimize effective drugs and new therapeutic protocols.

Our model contributes to a better understanding of *C. neoformans* metabolism, especially within the host environment. With this work, we propose new metabolic enzymes awaiting characterization and offer insights into key pathways and interactions shaping the dynamics between host and pathogen and its adaptive strategies. We also propose some potential antifungal targets for *C. neoformans* and confirm the coverage of already identified targets for that species. These results hold promise for the discovery of novel drug targets and the complete comprehension of this pathogen metabolic network with an expected impact in combating cryptococcosis.

## CRediT authorship contribution statement

**Diogo Couceiro:** Methodology, Investigation. **Romeu Viana:** Writing – review & editing, Writing – original draft, Visualization, Methodology, Investigation, Formal analysis, Data curation. **Luís Coutinho:** Writing – review & editing, Formal analysis. **William Newton:** Methodology, Investigation. **Carolina Coelho:** Writing – review & editing, Supervision, Methodology, Conceptualization. **Oscar Dias:** Methodology, Formal analysis. **Miguel Cacho Teixeira:** Writing – review & editing, Supervision, Methodology, Funding acquisition, Conceptualization.

## Author contributions

R.V., C.C., and M.C.T. conceived and designed the study. R.V. performed the model construction & development, data analysis and curation. R.V., D.C., and W.N. performed the experiments with *C. neoformans*. O.D. contributed to model construction and data analysis. L.C. performed data analysis. R.V. wrote the original draft preparation. R.V., L.C., C.C., and M.C.T. reviewed and edited the final version. All authors have read and agreed to the published version of the manuscript.

## Author statement

The authors declare no conflict of interest. The funders had no role in the design of the study; in the collection, analyses, or interpretation of data; in the writing of the manuscript, or in the decision to publish the results. Generative AI and AI-assisted technologies were not used in the writing process.

## Declaration of Competing Interest

The authors declare no conflict of interest. The funders had no role in the design of the study; in the collection, analyses, or interpretation of data; in the writing of the manuscript, or in the decision to publish the results.
